# Reflections of the social determinants of health on school children’s learning

**DOI:** 10.1590/1980-220X-REEUSP-2022-0345en

**Published:** 2023-01-23

**Authors:** Pamela Camila Fernandes Rumor, Ivonete Teresinha Schulter Buss Heidemann, Jeane Barros de Souza, Gisele Cristina Manfrini, Michelle Kuntz Durand, Richard Augusto Thomann Beckert

**Affiliations:** 1 Universidade Federal de Santa Catarina, Florianópolis, SC, Brazil.; 2 Universidade Federal da Fronteira Sul, Chapecó, SC, Brazil.

**Keywords:** Social Determinants of Healt, Child Healt, School Health Service, Underachievement, Determinantes Sociales de la Salud, Salud Infantil, Servicios de Salud Escolar, Rendimiento Escolar Bajo, Determinantes Sociais da Saúde, Saúde da Criança, Serviços de Saúde Escolar, Baixo Rendimento Escolar

## Abstract

**Objective:**

understanding the repercussions of the social determinants of health on school children’s learning, linked to the public-school network, from the point of view of health and education professionals.

**Method:**

qualitative study, participant-action type, articulated to Freire’s Research Itinerary, through three moments: Thematic Investigation; Coding and Decoding; Critical Unveiling. The participants were 27 health professionals and 18 education professionals, working in four municipalities of Santa Catarina. Individual interviews and a Culture Circle took place between November 2020 and March 2021. The analysis was developed in the discussions in the Culture Circle, with reflection on the themes.

**Results:**

unfavorable living conditions, unhealthy habits, and weak social relationships tend to negatively influence children’s school learning. There is a need for the implementation of actions involving the health and education sectors, such as the Health at School Program.

**Conclusion:**

the articulated action of health and education professionals on social determinants is the way to promote health and children’s school performance, in order to minimize the effects of existing inequities.

## RESUMO

**Objetivo:**

compreender os reflexos dos determinantes sociais da saúde na aprendizagem de crianças escolares, vinculadas à rede pública de ensino, sob a ótica dos profissionais da saúde e da educação.

**Método:**

estudo qualitativo, do tipo ação participante, articulado ao Itinerário de Pesquisa de Freire, percorrendo três momentos: Investigação Temática; Codificação e Decodificação; Desvelamento Crítico. Participaram 27 profissionais da saúde e 18 da educação, atuantes em quatro municípios de Santa Catarina. Realizaram-se entrevistas individuais e um Círculo de Cultura entre novembro de 2020 a março de 2021. A análise desenvolveu-se nas discussões no Círculo de Cultura, com reflexão dos temas.

**Resultados:**

as condições de vida desfavoráveis, hábitos pouco saudáveis e as relações sociais frágeis tendem a influenciar negativamente na aprendizagem escolar das crianças. Há necessidade de implementação de ações envolvendo o setor saúde e educação, como o Programa Saúde na Escola.

**Conclusão:**

a atuação articulada dos profissionais de saúde e educação sobre os determinantes sociais é o caminho para promover saúde e o desempenho escolar infantil, com vistas a minimizar os efeitos das iniquidades existentes.

## RESUMEN

**Objetivo:**

comprender los reflejos de los determinantes sociales de la salud en el aprendizaje de los escolares, vinculados a la red pública de enseñanza, desde la perspectiva de los profesionales de la salud y de la educación.

**Método:**

estudio cualitativo, de tipo acción-participante, articulado al Itinerario de Investigación de Freire, a través de tres momentos: Investigación temática; codificación y descodificación; desvelamiento crítico. Participaron 27 profesionales de la salud y 18 de la educación, que trabajan en cuatro municipios de Santa Catarina. Entre noviembre de 2020 y marzo de 2021 se celebraron entrevistas individuales y un Círculo de Cultura. El análisis se desarrolló en los debates del Círculo de Cultura, con reflexión sobre los temas.

**Resultados:**

las condiciones de vida desfavorables, los hábitos poco saludables y las relaciones sociales débiles tienden a influir negativamente en el aprendizaje escolar de los niños. Es necesario poner en marcha acciones que impliquen a los sectores sanitario y educativo, como el Programa Salud en la Escuela.

**Conclusión:**

la acción articulada de los profesionales de la salud y de la educación sobre los determinantes sociales es el camino para promover la salud y el rendimiento escolar de los niños, con el fin de minimizar los efectos de las desigualdades existentes.

## INTRODUCTION

Experiencing social inequities since childhood stages may be manifested by the difficulty or lack of several factors, such as access to public services and facilities, material and immaterial goods. There are factors that provide the reproduction of life in dignity, which interfere in the health conditions of individuals and in their development^(1)^. For full child development, the importance of education and health stands out, since together they provide the potential to fundamentally shape the trajectories throughout people’s lives, and education is pointed out as an important Social Determinant of Health - SDH^(2)^.

The SDH are the conditions under which people are born, live, grow, work, and age, corresponding to the social, economic, political, cultural, ethnic/racial, psychological, and behavioral factors that influence, affect, and condition the health of the population^(3)^. Given the social inequalities existing in Brazil, which includes income disparity, lack of access to goods and services, among others, it is considered that children in the public school network are a vulnerable group to several SDH, considered as indirect factors of the learning deficit^(4)^. Therefore, there is an urgent need for a broader look regarding the complexity surrounding the learning process, since children from disadvantaged backgrounds are more likely to have low school performance and, as adults, to have lower income and worse living and working conditions^(5)^.

There is a trend among teachers, schools, and other professionals involved in the learning process to blame children and their families for school failure, often reducing it to a supposedly individualized problem. This action is one of the reasons for the growing number of referrals of children with learning disabilities to health services, in an attempt to solve the situation under the medical perspective^(6)^. Learning difficulty is understood as any type of problem presented during the learning process, resulting from inherent or extrinsic conditions of the child, involving a broader context, either didactic-pedagogical and/or sociocultural^(7)^.

The fact is that poor school performance can bring consequences beyond the pedagogical dimension, such as psychological, behavioral, social, and repercussions beyond childhood^(8)^. Regardless of the etiology, Brazilian educational statistics indicate a challenging scenario, with high rates of failure, age/grade distortion, dropout, and evasion, resulting from poor performance due to non-learning in the first years of elementary school^(9)^.

The incidence of children with learning difficulties in the early years of elementary school has worsened the educational statistics, pointing to the high rates of unsatisfactory school performance in Brazil^(10)^. It is considered that learning the main skills, such as reading, writing, and calculating, have a positive impact on all life, being seen as an instrument to overcome social vulnerabilities and a condition for the full exercise of citizenship^(9)^.

Owing to the complexity that involves the learning process, it is necessary that the evaluation of difficulties be carried out taking into account not only changes in cognitive development, but also the contexts where children are inserted and the processes experienced by them^(10)^, which justifies this study. In view of these considerations, the question is: what are the reflexes of SDH on the learning of school children? It is urgent to shed light on this issue, since understanding SDH enables the search for appropriate intervention strategies in order to provide the necessary attention and minimize its harmful effect, especially because it is a risk factor for psychosocial problems in childhood, associated with socioemotional and behavioral problems^(11)^.

Therefore, it is imperative to elucidate the reality of these children and the SDH involved with this public, so that health and education professionals and managers can intervene on these factors, and thus promote actions to reduce the inequities to which they may be exposed, contributing to child development and well-being. Therefore, this study aimed to understand the effects of SDH on the learning of school children, linked to the public education network, as seen from the perspective of health and education professionals.

## METHODS

### Type of Study

This is a qualitative research, of the type action- participant^(12)^, based on Paulo Freire’s theoretical and methodological assumptions, which has as background a critical and liberating pedagogical proposal^(13)^. The Research Itinerary, which comprises three distinct and interconnected stages, was used: Thematic Investigation; Coding and Decoding; and Critical Unveiling^(14)^. The Research Itinerary considers the reality experienced by the subjects, inserted in a given social, historical and cultural context. This article was written according to the consolidated criteria for reporting qualitative research (COREQ).

### Study Setting

The study was developed in the contexts of health and education, in the cities of Florianópolis, São José, Palhoça and Biguaçu, which have the largest population contingent in the Macro-region of Greater Florianópolis, Santa Catarina. Regarding the settings, in the health care network, it took place at the primary level in each municipality, as this is considered the gateway to the Unified Health System (SUS), and in two clinical services specialized in attending school learning disabilities. Within the field of education, it involved four institutions in the basic municipal network, one state, and one federal, focusing on the initial years of elementary school.

### Population and Selection Criteria

Forty-five health and education professionals participated in the study. The professionals were chosen by convenience by the managers of each institution. Inclusion criteria were: health professionals working at the primary level and in clinical services specialized in treating learning disabilities in children aged six to ten years old; and education professionals in the early years of elementary school. Professionals who had been working for less than a year in the institution, or who were away on vacation or on leave during the data collection period were excluded from the study.

### Data Collection

The Research Itinerary was put in practice between November 2020 and April 2021. Due to the barriers imposed by the pandemic context of COVID-19, such as the need for social distance and the overload of activities in the health and education sector, the development of the stages of the Research Itinerary needed to be adapted. Firstly, individual semi- structured interviews were held with the 45 participants, in order to develop the first stage of the Freirean Itinerary, Thematic Investigation, in which the generating themes were raised. All the professionals who agreed to participate in the study were interviewed, with no emphasis on data saturation, in order to give voice to all the invited professionals who wanted to talk about the studied theme. In the second moment, a Virtual Culture Circle (VCC) was developed.

The interviews were previously scheduled, by telephone, and carried out in person or virtually, at the choice of each professional, being conducted by a doctoral student, and each of them lasted about an hour. It was supported by a script containing guiding questions about the learning difficulty at school and the relationship with the SDH of children and their families. It should be noted that before starting data collection, a pilot test of the interview was carried out with a representative of each selected location, having as a starting point the professionals’ perception of the SDH and the relationship with the teaching and learning process.

The face-to-face interviews were held in a room of each professionals’ institutions. With the interviews, a survey of the professionals’ personal and functional data was carried out, and the researchers prepared a chart, in a digital file, with words that reflected the generating themes extracted from the participants’ reality, thus constituting the Thematic Investigation.

The online interviews and the VCC were developed through the Google Meet application. For the development of the VCC, the professionals who participated in the first stage were contacted, but due to incompatible schedules and/or holidays and leaves, only 21 of them could be present, 10 from the health sector and 11 from education. The VCC was developed with a duration of two hours, mediated by one of the authors, a doctoral student, with the support of two facilitators with experience in conducting this type of approach.

In order to start the VCC, the themes raised in the interviews were projected onto the computer screen, validating with the participants their meanings, with a view to promoting the action and reflection process. For the Encoding and Decoding, the second step of the Research Itinerary, the mediator instigated the debate by dialoguing with the participants about the social factors that determined health and influenced children’s learning, relating them to the themes investigated. From this, two predominant themes were coded and decoded for discussion in the VCC, namely: I) Family living conditions; II) Child’s habits and social relations.

In the Critical Unveiling stage, the participants (re)signified the two generating themes and recognized the social factors that reflected on the children’s learning. At this moment, the real possibilities of transforming the reality experienced were discussed and, through a process of action-reflection, they socialized new perspectives to face these determinants in the context in which they acted. At the end, the mediator reread all the reflections that the group had built, in order to validate the data with all those involved in the VCC. In order to record the information, and with the due authorization from the participants, the interviews and the VCC were recorded, and later all the statements were transcribed.

### Data Analysis

The analysis of the themes occurred concomitantly with the development of the VCC, during the steps of Paulo Freire’s Research Itinerary, which provides this continuous analytical process, with the interaction of all participants^(14)^. Therefore, during the VCC, we counted on the participation of all those involved, researched and researchers, who, through dialogical praxis, exchanged experiences with each other, fostering a collective process of action and reflection. Thus, the social reality was critically unveiled, revealing what was hidden, therefore allowing the participants’ reflections to lead them to new proposals for action on the reality experienced, as suggested by Paulo Freire’s methodological approach.

### Ethical Aspects

The research followed the ethical principles of Resolution n. 466/2012, of the National Health Council, being initiated only after clearance by the Research Ethics Committee on the date of February nine, 2021, with opinion number 4.532.255. The Informed Consent Form was sent via e-mail to the participants, who, after reading and signing it, returned it via e-mail to the researchers. To ensure anonymity, names were replaced by the initials of the words in Portuguese for “Health Professionals” (PS) and “Education Professionals” (PE), followed by an Arabic numeral, for example: PS1, PE1, PS2, PE2, and so on.

## RESULTS

Forty-five professionals participated in the interviews, 18 from education and 27 from health. Among them, 41 were women and four were men, aged between 28 and 56 years. The participants belonged to different professional categories: five social workers; five nurses; three speech therapists; seven doctors; one dentist; 15 pedagogues; five psychologists; one psycho- pedagogue; one education technician; and two nursing technicians. In the education field: seven had undergraduate degrees; 31 specialized degrees; five had master’s degrees; and two doctorates.

Regarding the Health Care Network, 20 professionals represented primary care and seven the specialized clinical services for children with learning difficulties. In the Basic Education Network, five professionals belonged to the federal institution, five to the state institution, and eight to the municipal institution. The VCC had the participation of 10 health professionals and 11 education professionals.

In the first generating theme, family living conditions, the professionals talked about the importance of access to rights, goods, and essential services. These, when absent, place them in a situation of vulnerability, affecting mainly the most fragile members, such as children, which can have repercussions not only on their health, but can also bring damage to their school performance.

*The family has to have guaranteed rights, housing, food, infrastructure, sanitation, everything, for the children to be able to develop in its amplitude (…) And the child will not be able to stay in school, study and acquire knowledge if he/she is not healthy, his family is not healthy, because he suffers the consequences of the environment in which he/she lives*. (PE11)

They highlighted that the family socioeconomic situation is related to the financial possibility of acquiring resources and access to services that provide improvements for the child’s care, quality of life, as well as for its full school development. One of the most relevant factors mentioned by the professionals refers to food. They affirmed that many families do not have enough good quality food in their homes, and the problem of hunger is a reality experienced by many children.

*The food issue at the school is a very strong issue. There was a time when many students arrived with headaches in the (nursing) sector because they hadn’t eaten. It still happens, and then you ask, why didn’t you eat? Because they didn’t have anything to eat*. (PE9)

Regarding the housing conditions, they mentioned that there are dwellings in which there are few rooms and an extended family configuration, being considered unhealthy for child development. Thus, the unavailability of a suitable place to perform school tasks in these houses was pointed out as a limiting factor for the child’s study and learning.

*In our house, we have a table, a chair so we can sit and write. It may be that many of these families don’t have. I think that the lack of physical space can make it difficult for this child to do the activities* (PE8)

Regarding the level of education of the parents, the professionals mentioned that in families with less education or illiteracy, there is difficulty in access to information and knowledge about issues related to child development, as well as the importance of stimulating the learning of their children.

*Not all children who come to school have gone through pre-school. Many of them just stayed at home, without any stimulation. There are parents who can barely read, so it is difficult, because they (the children) don’t have the support of an adult who can guide them, help them with their tasks*. (PE15)

In relation to the access to child health care, they revealed that children generally do not have regular monitoring of growth and development, especially those who depend exclusively on the public network.

*Many children lose contact with the health unit after the four-year vaccine calendar (…). It is a big failure in primary care not to privilege this follow-up. It would be fundamental to have a global evaluation of this child, physical, mental, vision, hearing and language health*. (PS27)

In the second generating theme, habits and social relations of the child, the participants listed some aspects they considered important to keep children healthy and favor their school performance, such as proper nutrition, physical exercise and leisure.

*First, a balanced diet is something basic, and the practice of some physical activity is fundamental. Leisure activities, doing the things you like, to feel happy* (PE10)

In addition, they stated that there are few recreational opportunities for children for physical and playful activities outside the school context. Children’s time has been filled by long periods in front of screens.

*The father, mother or guardian has to work the whole day, most of the time. The child, let’s say, comes to school in the morning, in the afternoon, he/she doesn’t have a sport, an activity, he/she stays home alone (…) The next day, they arrive at school tired, sleepy, because they stayed too late on TV*. (PE2)

For the professionals, the absence of a routine, with established schedules, care performance and parental supervision, is also a factor that harms health and school learning.

*I realize that it is a little difficult for the family to organize itself. In my time, I had a time to play, to eat, I had a time to study, to go to school. Today, families have no routine, and children need this.* (PS18)

The structure and the good coexistence in intra-familial relationships were mentioned as primordial elements for child development, especially affective-social, with relevant importance in learning. However, the existence of conflicts and broken family bonds were considered determinants that can lead to the occurrence of emotional and behavioral changes, which reflect on school performance.

*If the child is not doing well, if things happen in his life that are not good and that affect him, like being in a toxic environment, an environment that is heavy, has many fights, this is bad, both mentally and psychologically. How will he/she learn? He/She doesn’t care, there are other things that make him/her sad*. (PE17)

They also highlighted the parents’ emotional support to the child, which can be characterized either by lack of attention and affection, or even by overprotection and exacerbated expectation for their children’s school performance, which affects not only their psychological but also the evolution of their learning.

*I think that sometimes the family doesn’t give the child a chance to advance, it’s an overprotective mother, or a mother who demands too much, or who doesn’t give as much attention, so this also changes the child’s performance*. (PS26)

Another fundamental support network pointed out by the professionals was the school, considered an important place for social interaction, which greatly contributes to the formation, development, and learning.

*The child who is in the learning process needs to feel welcome, he needs to feel safe at school. We have to praise and extol the successes of the child, and not only highlight the mistakes*. (PE3)

The participants mention the existence of stigmas in the school context, especially about children with learning difficulties, with a tendency to social prejudice and creation of labels associated with this demand, which are inclined to underestimate them, with demonstrations of bullying/cyberbullying.

*I did some work in a school on behalf of the Health in the School Program, and I was struck by the issue of bullying. How present it is in the school, almost as a reality that is part of the school context. It is really very worrying and little noticed. Not only bullying, but cyberbullying that has a reach, a much larger projection, and really, emotionally, affects mental health, and has repercussions on the learning process*. (PS8)

It was discussed that there are barriers that are encountered in the dynamics of the work process, such as the very structure of the education network.

*We have several complexities, besides the whole network structuring that needs to be discussed* (…) (PE14)

The participants tried to redefine and propose changes in their reality. They revealed that it is necessary to strengthen intersectoral actions for a more effective action on the determinants.

*Regarding what we may have control, I think the main way is intersectoriality, is to maintain a communication channel among professionals*. (PS7)

They talked about the intersectoral work of the Health in the School Program, which is based on the articulation between the school and the basic health network, with the possibility of participation by other sectors.

*The Health in the School Program is a perspective where we identify how the influence of these social determinants can occur in this issue of children’s learning (…). It would be very interesting, at the local level, and sometimes even at a more macro level, for us to think about forums for intersectoral articulation, among these various public policies (education, health, and social welfare), because then we could add (…) stop the issue of school learning difficulty with these numerous determinants that end up influencing it*. (PS15)

For professionals, the Health in the School Program has the potential to articulate and coordinate actions on behalf of children’s health, considering the SDH involved and its effects on children’s learning, because it provides greater integration and strengthening among services and professionals.

## DISCUSSION

Seen from the perspective of SDH, health and all human development are influenced by events to which people are exposed throughout life. There are events that are potentially adverse, originating from the social and economic precariousness in which many families live, as highlighted in the first theme that generated the results of this study, with direct impact on different domains - physical, cognitive, emotional, occupational^(15)^. Poor living conditions, unfavorable habits, and fragile social relationships are factors that determine the health of children and are related to the issue of school difficulty, even indirectly, given the negative interference they cause in the child learning process^(7)^.

The conditions in which children live are significant aspects in the development of their capabilities, such as learning, and their way of being and knowing the world, shaping their life habits^(5)^. It must be considered that living conditions have improved continuously and are sustained in most countries thanks to political, economic, social, environmental progress and advances in public health. The social inequalities existing in the Brazilian context affect a large portion of the population and are evidenced by the unfair distribution of income, as well as in the scarce -or nonexistent- opportunities for economic and social inclusion, impacting the health and educational situation of children^(4)^.

Poverty severely reduces the chance to live a healthy life, and both historically and globally, it has been the main direct and indirect cause of poor health and social inequities^(16)^. It is conceived that a high family socioeconomic position enables recovery from adverse events suffered by the child, or even protects from their occurrence. Regarding education, the participants of this study reflected that the lower the family income per capita and the parents’ education, the greater the occurrence of learning difficulties and repetitions^(17)^. Education promotes individual development and enables conditions to create projects that improve health conditions and to develop the living environment^(18)^. In families with parents with higher educational level, children are more likely to have a healthy cognitive development and consequently, educational advantage and greater access to information^(19)^.

In this sense, educational success is not equally distributed in society, since often, it is people living in disadvantaged circumstances who have poor educational performance and less access to good quality educational services^(16)^. The process of their children’s schooling tends not to be valued by parents, since their lives have not been transformed by the educational process, as highlighted by the participants of this study. It is important to understand that many of these parents were also children who presented difficulties at school, due to lack of access -or the need to work from an early age- being therefore victims of a society of few opportunities and violation of essential rights^(15)^.

Another factor to ponder is that the lack of financial resources for the family’s subsistence tends to bring other consequences, such as the deprivation of access to food. Food needs were intensely addressed by the interviewees in this study as a factor that reflects on the children’s school learning. It has a central position in learning and social formation, and the provision of meals at school is a key strategy for contributing to the biopsychosocial development, performance and permanence of children at school^(20)^.

Families with precarious living conditions tend to have impaired access to good-quality housing, and housing conditions involve an important determinant of health, contributing to the well-being and human dignity of its members^(21)^. Moreover, there is a close interface between housing conditions and school performance, since children who live in precarious places are more likely to have their performance compromised^(22)^, as discussed by professionals in the dialogues in the VCC.

It is noteworthy that child health is a direct reflection of family socioeconomic conditions, and the existing inequities often make it impossible or difficult to access health services, treatments, and technologies^(1)^. The Family Health Strategy has favored the access and surveillance of children’s health, although there are still regional disparities in the coverage of the primary care network in Brazil, with little guarantee of comprehensive care and predominance of curative actions. Such inadequacies can lead to inability to meet the health needs of this population and the non-recognition of the importance of periodic monitoring of children in primary care, as evidenced by the professionals in this study, who reported that such monitoring is usually performed only with regard to vaccination in early childhood^(23)^.

Lifestyle factors involve individual and family responsibilities, which depend on people’s choices, being DSS that can lead to variable healthy habits, as discussed in the second theme that generated the results of this research. Associated with this, situations of vulnerability can open gaps for families, whose formal networks of protection are not at reach, to adopt inappropriate lifestyles that may bring bad outcomes for the child’s health, with repercussions throughout their life cycle^(4)^.

Since childhood is a phase marked by the formation of eating habits, the provision of a healthy diet, rich in nutrients, is essential for biopsychosocial growth and development, which also favors the acquisition of learning^(24)^. Similarly, the respondents of this study also stressed the importance of the adoption of physical activities by children, which play a key role, especially in cognitive, intellectual and motor functions, contributing to children’s school performance, among other benefits^(25)^.

When reflecting on the children’s learning deficit, it is important to consider the SDH, since it is postulated that not only the constitutional factors and individual lifestyle impact people’s health, but also the social and community networks, meeting the considerations made by the participants of this study^(26)^. The family is the child’s initial social network, essential to ensure the survival and full protection of children, regardless of how it has been structured. It provides the construction of affective bonds and the satisfaction of needs in the development of the person, plays a decisive role in socialization and education, with organization of the children’s routine in their daily lives. For a healthy learning process, it is necessary that the family context provides conditions for this, since events within the family, such as conflict, violence, lack of emotional support, can negatively affect the child’s cognitive development^(27)^.

The first social bonds are formed in the school context, which is a space for socio-educational training, capable of contributing significantly to the formation of people in a full, integral, and healthy way. The importance of the teacher’s role stands out, because when well trained and sensitive about the learning and development processes of schoolchildren, he/she acts both in the promotion of mental health and in the prevention of difficulties, seeking to interrupt the logic of “pathologizing” the field of learning^(26)^. In this sense, it is necessary to overcome the historical production of violence in the school environment, including the practice of bullying and cyberbullying, as highlighted in this study by health and education professionals, which exposes many children to the condition of vulnerability, interfering in the teaching-learning process and in the student’s health^(28)^.

After analyzing these considerations, in order to overcome the barriers established in childhood and strengthen the foundations of health and lifelong learning, it is noted that in the dialogues arising from this study, there is a concern with SDH and its relationship to child learning. Beyond this, there is a pressing need for more effective strategies from services and public policy to support children’s health and development by addressing poverty, housing instability, food insecurity, and other sources of adversity that impose significant stresses on families.

Although there is consensus on the importance and global interest in the theme, the process of implementing approaches related to SDH to reduce inequities and improve health status has been slow and fragile, especially in developing countries, where barriers imposed by disadvantage and social injustices predominate^(11)^. It is considered that integrated actions, built in an intersectoral way, may be the key to a new dynamic in the governmental devices and to the search for a more equitable society, constituting a modality of interference in problems in the field of social practices^(29)^.

The health promotion movement has collaborated in the confrontation of social inequities, constituting a relevant support for the implementation of crosscutting policies^(30)^. For this reason, and with regard to school health, as well stated by the interviewees of this study, the Health in the School Program has proved to be a tool for addressing the vulnerabilities that compromise the full child development, with a view to promoting health and, consequently, the learning of children from the basic public education network, besides contributing to the improvement of living habits^(5)^.

As limitations of this study, we mention the restrictions imposed by the pandemic context, which initially generated discomfort due to the need to adapt the Research Itinerary to the virtual format. However, the VCC was configured as an effective possibility for carrying out participant action-research in pandemic times, with the involvement of different professional categories and sectors, with simultaneous integration of geographically distant people, providing a space for the exchange of knowledge in a conscious and critical action about the studied phenomenon.

## CONCLUSIONS

This study allowed us to recognize that SDH reflect on children’s school learning, involving several factors, such as: precarious living conditions; exposure to unfavorable habits; weakening of social networks, especially because of the social vulnerability situation to which many families of schoolchildren in the public school system are exposed.

It is essential to elaborate and implement public policies that encompass several sectors, especially education and health, that recognize the importance of healthy child development and full educational performance, especially through interprofessional action, as potentials for reducing the existing social inequities in Brazil.

In order to continue contributing to the universe of health in the school, we recommend other studies with families, professionals, and managers, including different sectors, in order to deepen the knowledge about the learning difficulty of children. Through further investigations, it is believed that the possibilities for acting on the SDHs that reflect on the learning of school children can be expanded.
